# Supplementary material for: Clinical outcome prediction from analysis of microelectrode recordings using deep learning in subthalamic deep brain stimulation for Parkinson`s disease
Source: PLoS One. 2021 Jan 26;16(1):e0244133. doi: 10.1371/journal.pone.0244133 (PMC7837468; doi:10.1371/journal.pone.0244133)
Supplement: S1 Material — (DOCX) [file pone.0244133.s001.docx]

## S1 Material.

## Hardware Specifications

CPU: Intel core i7-7700 3.60Hz x 8

GPU: TITAN X (Pascal) 12GB

RAM: 16GB

## Software Specifications

Deep learning libraries

Tensorflow – 1.14.0 with cuda 10.0 and cudnn 7.6.3

Python libraries (version - 3.6)

Numpy – 1.16.4 for array processing

Pandas – for excel signal load

Scipy(Signal) – 1.1.0 for signal filtering

Matplotlib – 3.1.1 for plotting signal as image

Os – for filename load

Obspy signal – Continuous Wavelet Transformation Function (21)

Optimizer - GradientDescentOptimizer – learning rate of 10 e^-3^
