# Supplementary material for: Clinical outcome prediction from analysis of microelectrode recordings using deep learning in subthalamic deep brain stimulation for Parkinson`s disease
Source: PLoS One. 2021 Jan 26;16(1):e0244133. doi: 10.1371/journal.pone.0244133 (PMC7837468; doi:10.1371/journal.pone.0244133)
Supplement: Attachment — Submitted filename: Response to Reviewer_3.docx [file pone.0244133.s002.docx]

**Reviewer #3**

The authors report on the clinical prediction of the clinical outcome of DBS using deep learning. While the main idea is interesting, the study requires substantial revision. The main criticism is due to the fact the authors did not experiment with different machine /deep learning algorithms to find an algorithm that performs well. In conclusion, the authors mention the following

*“We expected to be able to use machine learning to determine the differences that cannot be found in a conventional way. However, it is not possible to accept the result because of various limitations. After correcting the limitations one-by-one and satisfying the results, we will be able to determine the hidden factor in the MER data.”*

This statement which aims to summarize the finding, shows that the study does not add much to the reader, because finding the right ML/DL tool and/or performing extensive parameter search to obtain the right algorithm is the main task when one hopes to employ DL/ML. By design, the ML/DL algorithms require fine-tuning to achieve the best outcome. Hence I recommend that the authors attempt to either modify the convolutional Neural network they use or look into other types of ML/DL tools that provide a satisfactory prediction.

- We understand the limitation. However, we think it is of great significance to apply end-to-end deep learning and to present the beginning of a new analysis method that utilizes CNN and multitask learning in the signal analysis.

I have to add that the presentation and the flow of the text needs improvement as well. The paragraphs do not follow well, and at times the reader needs to think hard to understand the transition.

In the following, I also have some more comments which I hope the author can take into account in the next version:

1. The title of the paper needs to be modified. As of now, one struggle to read it because of long compound nouns “deep learning-based microelectrode recording analysis” and “subthalamic deep brain stimulation” I recommend something as follows:
   Analysis of microelectrode recordings from Deep brain stimulation using Deep learning or Leveraging deep learning for analysis of microelectrode recordings from Deep brain stimulation. Long nouns statements make the title hard to read and are not recommended by the English language educators (For more see “Williams, Joseph M., and Joseph Bizup. *Lessons in clarity and grace*. Pearson, 2014.”)

🡪 We agree with you. We will change the title to “Clinical outcome prediction from analysis of microelectrode recordings using deep learning in subthalamic deep brain stimulation for Parkinson`s disease”

2. Do not define any acronyms in the abstract. The reader won’t remember them and one has to go back many times.

🡪 We understand what you are worrying. However, if we don't use acronyms, we are concerned that the abstract will be too long to understand. For most stereotactic and functional neurosurgeons, those acronyms such as PD, DBS, MER, UPDRS are commonly used. Some other acronyms such as VGG, AUC, ROC were used to help understanding, because they are familiar then the full name.

3. The abstract needs to be a coherent text, I recommend combining all 4 parts. One can avoid stating the method in detail; just mention the goal of the study and state the outcomes. The purpose of an abstract is to spark the reader’s interest in the story and motivate them to continue reading the whole paper. An easy-to-follow and compelling writing style and story-telling is needed in the abstract. Also, diving into the details of the method in the abstract may not be the best.

🡪 We agree with your purpose. We made some changes to abstract to make it easier for readers to understand and find interest. It is impossible to delete more basic information from the method, because the novel point of this article is the methods.

4. Citation for relevant literature is missing in the introduction; make sure please review the relevant DBS literature in the introduction. The authors need to guide the reader ensuring that the reader can understand the significance of the issue by providing a coherent and cohesion text.
   a. The text in the introduction jumps between topics and makes it rather hard to follow. For instance in line 12-127 the authors suddenly jump from discussing PD to Convolutional Neural Networks. This qualifies as a sharp and unexpected turn which leaves the readers baffled for a second. See the highlighted text below please
   *“...no studies have deep learning-based analyze MER fsignals with the clinical outcomes of patients with advanced* ***PD after STN DBS. Convolutional neural network (CNN) models can process*** *signals using imaging processing methods”*
   The authors need to make a case for the specific tool and provide smooth transitions. Also when discussing CNN, one has to show why it is the best tool for this task? As we can see later on the text CNN is not the best tool!

🡪 We totally agree with you. That sentence was duplicated in the wrong paragraph by mistake. The next paragraph is the smooth transition you mentioned. Thank you.

5. At the end of the introduction please outline the study so the reader knows is coming next.

🡪 We revised last paragraph.

6. Only off-medication status was included in this study. It would be informative to consider both on and off medication.

🡪 We understand your opinion. We thought off-medication status was the most important point of the outcome of the surgery. But we are now looking for the way to consider other conditions such as age, BMI, DM, postoperative events. However, we think it is of great significance to apply end-to-end deep learning and to present the beginning of a new analysis method that utilizes CNN and multitask learning in the signal analysis.

7. Do the authors did any sort control group to see whether CNN performs better or any different on the data from that group? If not, it would be useful to have a control group. Also before using CNN on the MER data from the patients, do we have know indication that CNN will perform well on the MER data? Simply assuming CNN will work on this data because it has worked on other data is not the best argument.

🡪 We totally agree with the reviewer’s comment on control group. To feed the raw signal to the ML algorithm, the sampling rate of the signal is large and there are few well known features about MER. So we used image based CNN method. We have added sentences to limitation session.

“Since the sampling rate of the signal is large and there are few well known features about MER. So we could not have a better machine learning control group.”

8. The authors train CNN using data from four patients; and used this to predict the outcome for other patients; This could be the source of possible error and the reason for the poor performance in the prediction. Maybe one can use data from each patient to train a patient-specific CNN. Why would a CNN trained with data from four patients should be able to predict the outcome for all other patients?

🡪 We totally agree with the reviewer’s comment on performance prediction. CNN was trained with thirty patients and test was done by four patients. Four patient was chosen randomly. And we have considered about personalized model but universal training can predict MER signal without using the previous signal. We have added sentences to limitation session.

“Personalized model was considered but universal training can predict MER signal without using the previous signal”

9. In the Deep learning section, one has to present more than a general knowledge and dive deep into why the DL is useful, and talk in depth about the tool used, why it is the best, and the reason behind the choice of the parameters for the DL algorithm. From the text one might think RNN and CNN are the only artificial neural networks.

🡪 We totally agree with the reviewer’s comment general knowledge about deep learning. We added several sentences to methods section.

“CNN extracts features from images using convolution and pooling layers, extracted features were classified with fully connected layers”

10. I recommend moving the software/hardware and and the statistical tools specification to the supplementary material, maybe one can include a code; most certainly a new section is not required for each specification.

🡪 We totally agree with the reviewer’s comment about software/hardware and moved to supplementary material. We moved following hardware and software specifications to Supplementary material.

11. The results section needs to be extended and provide a detailed explanation of the results; simply putting the figures; is not enough .

🡪 We revised some paragraphs of the result section.

12. In lines 361-362 references are needed for this statement “which is consistent with other papers that reported clinical outcomes in unilateral DBS”

🡪 We totally agree with you. It is revised.

13. In Fig 13 where the authors present the accuracy for test and training data, the poor performance in the test data is an indication of the poor learning. The accuracy of around 0.5, is not statistifactoty and one has to One can do an extensive parameter search, and/or try different algorithms to and find something suitable for the given problem.

🡪 We totally agree with the reviewer’s comment about optimization of control group. Various optimizers were adopted to converge the CNN, but the validation accuracy did not increase. We added several sentences to limitation section.
“Various optimizers were adopted to converge the CNN, but the validation accuracy did not increase in control group.”

14. The figures need to be combined so the reader will look at one figure instead of many that present similar data, also teh captions could be extended to make sure the reader can understand what is presented in the figure without having to go back to the text.

🡪 We agree with you. We revised some captions.
