# Supplementary material for: Clinical outcome prediction from analysis of microelectrode recordings using deep learning in subthalamic deep brain stimulation for Parkinson`s disease
Source: PLoS One. 2021 Jan 26;16(1):e0244133. doi: 10.1371/journal.pone.0244133 (PMC7837468; doi:10.1371/journal.pone.0244133)
Supplement: Attachment — Submitted filename: Response to Reviewers.docx [file pone.0244133.s003.docx]

**Response to Reviewers**

1. If the authors have adequately addressed your comments raised in a previous round of review and you feel that this manuscript is now acceptable for publication, you may indicate that here to bypass the “Comments to the Author” section, enter your conflict of interest statement in the “Confidential to Editor” section, and submit your "Accept" recommendation.

Reviewer #2: All comments have been addressed

Reviewer #3: (No Response)

2. Is the manuscript technically sound, and do the data support the conclusions?

The manuscript must describe a technically sound piece of scientific research with data that supports the conclusions. Experiments must have been conducted rigorously, with appropriate controls, replication, and sample sizes. The conclusions must be drawn appropriately based on the data presented.

Reviewer #2: Yes

Reviewer #3: Yes

3. Has the statistical analysis been performed appropriately and rigorously?

Reviewer #2: Yes

Reviewer #3: Yes

4. Have the authors made all data underlying the findings in their manuscript fully available?

The [PLOS Data policy](http://www.plosone.org/static/policies.action#sharing) requires authors to make all data underlying the findings described in their manuscript fully available without restriction, with rare exception (please refer to the Data Availability Statement in the manuscript PDF file). The data should be provided as part of the manuscript or its supporting information, or deposited to a public repository. For example, in addition to summary statistics, the data points behind means, medians and variance measures should be available. If there are restrictions on publicly sharing data—e.g. participant privacy or use of data from a third party—those must be specified.

Reviewer #2: Yes

Reviewer #3: Yes

5. Is the manuscript presented in an intelligible fashion and written in standard English?

PLOS ONE does not copyedit accepted manuscripts, so the language in submitted articles must be clear, correct, and unambiguous. Any typographical or grammatical errors should be corrected at revision, so please note any specific errors here.

Reviewer #2: Yes

Reviewer #3: No

6. Review Comments to the Author

Please use the space provided to explain your answers to the questions above. You may also include additional comments for the author, including concerns about dual publication, research ethics, or publication ethics. (Please upload your review as an attachment if it exceeds 20,000 characters)

Reviewer #2: I have no current comments except for the inclusion of the safety issues in the future works, which I had previously clarified in the previous review.

- Thanks for your comment. This study was restrospective study. In future research, we will consider safety issues.

Reviewer #3: This is an interesting and novel proposal to predict postsurgical results. In this retrospective study you have recollected a valuable dataset and the statistical analysis has been run appropriately. In general terms, the written English, the prose of the manuscript, and the medical terminology leaves much room for improvement and at times, perhaps, language translation errors play a roll in this manuscript. I highly recommend a complete review of the prose and the medical terminology utilized to narrate the background, findings and results. In terms of the usage of numbers in their numerical format versus written format, I suggest developing a consistent and singular style throughout the manuscript. In my opinion, the narrative will reach the elegant and professional level require if a native English speaker scientist help to re-write this manuscript.

- At the time of the first submission, I received English correction from a professional proofreading company. This revised version was now also received English correction. Attach the certificate as well.
- We did complete review of prose and the medical terminology with the help of a professional translation correction company.

Please indicate the co-author Young Hoo Lim degree.
🡪 Thank you. It is revised.

The scales numbers in figure#1 are illegible.

🡪 Sorry, but the scale in the figure is not important, because it was just one example of the frequency ranges. And the scale is same as figure 6A-F

Abstract

A lot of time and some assumptions was necessary to understand the ideas that were intended to describe. There is a lack of connection between the correct engineering and clinical terminology. Likewise, no details that complete and concrete the evidence previously publish is mentioned across the whole abstract. I recommend consider the possibility to re-write it.

🡪 It is modified. We added some details.

For example, in line 88 (the objective section) you start the paragraph saying what you did, is not clear what are the outcomes expected. You wrote: to better predict motor function improvement; motor function is a general term, are you referring to tremor? Rigidity? Bradykinesia? Postural instability?

🡪 motor function means UPDRS part III scores including rigidity, bradykinesia, postural instability.

In line 90, you wrote: we could make the outcome better even under restriction. What is the outcome?
🡪 That means UPDRS part III score. It is modified.

The methods section is very confusing; therefore, the results are unclear.
🡪 It is modified. We added some details.

Introduction

In the introduction (line 122-123) you mentioned the definition and targeting of the STN has been improved due to advances in magnetic resonance imaging (MRI) techniques. Are you referring to the accuracy of in vivo identification and localization of the brain anatomical structures in behalf of the resolution improvements in MRI (3T or 7T)? If this is relevant as a background in this study, can you provide reference?

- Yes. We added the reference.

Also each line of the introduction require some adjusted like the above.

Dear author, in this point, I congratulate you for the innovation and the effort putting this date together. This is a valuable date and deserve an elegant and well explained presentation, respectfully, I suggest re-write the manuscript and re-submit to this journal again.

7. PLOS authors have the option to publish the peer review history of their article ([what does this mean?](https://journals.plos.org/plosone/s/editorial-and-peer-review-process#loc-peer-review-history)). If published, this will include your full peer review and any attached files.


If you choose “no”, your identity will remain anonymous but your review may still be made public.


**Do you want your identity to be public for this peer review?** For information about this choice, including consent withdrawal, please see our [Privacy Policy](https://www.plos.org/privacy-policy).

Reviewer #2: **Yes:** MM

Reviewer #3: **Yes:** Cinthya Aguero

[NOTE: If reviewer comments were submitted as an attachment file, they will be attached to this email and accessible via the submission site. Please log into your account, locate the manuscript record, and check for the action link "View Attachments". If this link does not appear, there are no attachment files.]

While revising your submission, please upload your figure files to the Preflight Analysis and Conversion Engine (PACE) digital diagnostic tool, <https://pacev2.apexcovantage.com/>. PACE helps ensure that figures meet PLOS requirements. To use PACE, you must first register as a user. Registration is free. Then, login and navigate to the UPLOAD tab, where you will find detailed instructions on how to use the tool. If you encounter any issues or have any questions when using PACE, please email PLOS at [figures@plos.org](mailto:figures@plos.org). Please note that Supporting Information files do not need this step.
